# Sub-picosecond temporal resolution of anomalous Hall currents in GaAs

**DOI:** 10.1038/s41598-017-11603-4

**Published:** 2017-09-11

**Authors:** Christian B. Schmidt, Shekhar Priyadarshi, Mark Bieler

**Affiliations:** 1 Physikalisch-Technische Bundesanstalt, Bundesallee 100, 38116 Braunschweig, Germany; 2 Present Address: Leibniz Institute of Photonic Technology, 07745 Jena, Germany; 3 Present Address: Institute of Physical Chemistry, University of Hamburg, 20146 Hamburg, Germany

## Abstract

The anomalous Hall (AH) and spin Hall effects are important tools for the generation, control, and detection of spin and spin-polarized currents in solids and, thus, hold promises for future spintronic applications. Despite tremendous work on these effects, their ultrafast dynamic response is still not well explored. Here, we induce ultrafast AH currents in a magnetically-biased semiconductor by optical femtosecond excitation at room temperature. The currents’ dynamics are studied by detecting the simultaneously emitted THz radiation. We show that the temporal shape of the AH currents can be extracted by comparing its THz radiation to the THz radiation emitted from optically induced currents whose temporal shape is well known. We observe a complex temporal shape of the AH currents suggesting that different microscopic origins contribute to the current dynamics. This is further confirmed by photon energy dependent measurements revealing a current inversion at low optical excitation intensities. Our work is a first step towards full time resolution of AH and spin Hall currents and helps to better understand the underlying microscopic origins, being a prerequisite for ultrafast spintronic applications using such currents.

## Introduction

Employing the spin degree of freedom for information processing enables promising new applications^1, 2^. To connect such spintronic techniques with conventional electronics, it will be essential to transform spin information into charge information. This can be realized using the spin Hall^3–5^ and inverse spin Hall^6, 7^ effects, converting charge currents into spin currents and vice versa, respectively. Both effects result from the microscopic mechanisms being also responsible for the anomalous Hall (AH) effect, which denotes a deflection of carriers into an apparently “anomalous” direction. These microscopic mechanisms are either of extrinsic (scattering effects) or intrinsic (Berry curvature) nature^8–11^.

The AH Hall effect requires a broken time-reversal symmetry and is a consequence of spin-orbit coupling. While this effect has been known to exist in ferromagnetic materials since the 19^th^ century, first measurements of AH currents in semiconductors have been performed in 1984 by Bakun *et al*.^12^, where the time-reversal symmetry was broken by excitation of the semiconductor with circularly-polarized light. Two decades later, the topic gained strong interest again, mainly via experimental and theoretical studies of the (inverse) spin Hall effect^3–7, 13, 14^ allowing for numerous spintronic applications and it was soon recognized that the AH effect and spin Hall effect share the same microscopic origins^1, 2, 9^. The intrinsic contribution results from the Berry curvature^8, 9^, which resembles a magnetic monopole in momentum space and only depends on momentum-space derivations of the Bloch functions. In contrast, the extrinsic contribution appears due to scattering of carriers with impurities or phonons and can be divided into three different components, referred to as side jump, skew scattering, and anomalous carrier redistribution^10, 15^. Yet, despite numerous studies, full insight into the interplay between intrinsic and extrinsic mechanisms has not yet been accomplished^2, 9, 10^. As recently demonstrated, time resolved measurements of the anomalous velocity^16^ might be a possibility to distinguish between intrinsic and extrinsic effects and to gain new insight into the underlying microscopic origins, calling for additional time-resolved studies.

Here, we optically induce AH currents in GaAs and detect the THz radiation emitted from the currents with a sub-picosecond time resolution. Since the shape of the THz traces is altered by propagation and detection effects, it is not possible to directly obtain the dynamics of the AH currents from THz measurements. However, the AH currents’ temporal shape can be estimated by comparing its THz emission with THz emission from reference currents whose temporal shape is well known. Taking advantage of this approach we demonstrate that the AH currents possess a complex temporal shape, which depends on excitation intensity and photon energy. At low excitation intensities a current inversion occurs for different excitation photon energies. This directly shows that different microscopic effects compete and influence the AH current dynamics.

## Anomalous Hall Currents

**Figure 1:**
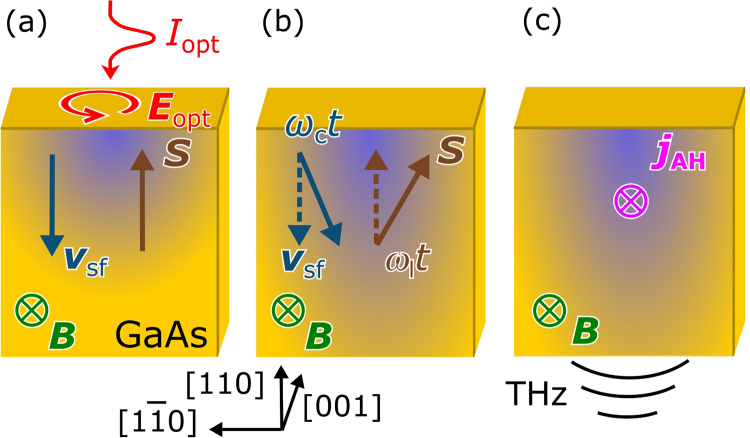
Generation of AH currents. (**a**) Optical excitation creates spin alignment (**S**). The spins move (${{\bf{v}}}_{{\rm{sf}}}$) in the surface electric field. (**b**) A static in-plane magnetic field (**B**) induces Lamor precession and Hall rotation of **S** and ${{\bf{v}}}_{{\rm{sf}}}$, respectively. (**c**) The AH current along the direction ${\bf{S}}\times {{\bf{v}}}_{{\rm{sf}}}$ emits THz radiation which is detected with a sub-picosecond time resolution. It should be noted that the generation of AH currents cannot be accomplished for an out-of-plane magnetic field since in this case **S** and ${{\bf{v}}}_{{\rm{sf}}}$ are parallel to **B** and, consequently, no spin Hall angle between **S** and ${{\bf{v}}}_{{\rm{sf}}}$ will be formed.

We first introduce details of AH current generation in GaAs. Our measurement scheme as shown in Fig. 1 is identical to previous experiments^17^, in which surface and bulk contributions to magneto-optically induced currents were separated. Moreover, the geometry is similar to the one used by Bakun *et al*.^12^, just that the generation and detection of the AH current are accomplished in a time-resolved manner. The process can be separated into three steps as shown in Fig. 1. First, we employ circularly polarized femtosecond laser pulses being normally incident on the GaAs sample to excite carriers with spin (**S**) arranged parallel or antiparallel (depending on the helicity of the circular polarization) to the optical wave vector. The spin polarized carriers move with velocity **v**_sf_ due to the spatial spin gradient and the surface electric field with the latter dominating for intrinsic bulk GaAs for the photon energies used here^18^. Second, an applied in-plane magnetic field (**B**) rotates the carrier spin (Lamor precession) and the spin flow (Hall rotation) creating the so-called spin Hall angle between spin and spin flow. Third, the spin Hall angle leads to the AH current (**j**_AH_) in a direction given by ${\bf{S}}\times {{\bf{v}}}_{{\rm{sf}}}$ ^2, 6^. Thus, a spin component perpendicular to the velocity is required for AH current generation. Only in this geometry the Berry curvature and the scattering centers interact with the spin to modify the direction of motion of the carriers and lead to AH currents.

Having introduced the AH currents, we like to comment on the terminology. The described current generation process does not involve pure spin currents but only spin-polarized charge currents being deflected along an “anomalous” direction, as it is the case in anomalous Hall measurements. Therefore, we believe that it is more appropriate to refer to the currents under study as AH currents instead of inverse spin Hall currents or similar expressions.

Since the optical excitation and the subsequent current dynamics occur on a femtosecond time scale, electromagnetic THz radiation (${{\bf{e}}}_{{\rm{AH}}}$) is emitted, which is detected with a sub-picosecond time resolution using electro-optic sampling (EOS), see methods. The THz traces are proportional to the time derivative of the currents convolved with the transfer functions^19^ of THz propagation, ${{\bf{H}}}_{{\rm{prop}}}$, and detection, ${{\bf{H}}}_{{\rm{EOS}}}$, see methods:

1 $${{\bf{E}}}_{{\rm{A}}{\rm{H}}}\propto {{\bf{H}}}_{{\rm{p}}{\rm{r}}{\rm{o}}{\rm{p}}}{{\bf{H}}}_{{\rm{E}}{\rm{O}}{\rm{S}}}i\omega {{\bf{J}}}_{{\rm{A}}{\rm{H}}}.$$

Here and throughout this paper upper-case letters (e.g., ${{\bf{J}}}_{{\rm{AH}}}$) denote the Fourier transformation of the time-domain variables expressed by lower-case letters (e.g., ${{\bf{j}}}_{{\rm{AH}}}$) with the frequency and time dependence being taken as implicit. Both transfer functions, ${{\bf{H}}}_{{\rm{prop}}}$ and ${{\bf{H}}}_{{\rm{EOS}}}$, will significantly change the time-domain shape of the THz traces^19, 20^. While some spectral information of ${{\bf{J}}}_{{\rm{AH}}}$ will be lost due to the multiplication of ${{\bf{J}}}_{{\rm{AH}}}$ with the transfer functions, see Eq. (1), and cannot be recovered, the time-domain shape of ${{\bf{j}}}_{{\rm{AH}}}$ could at least be estimated, if the transfer functions were known. However, the accurate simulation or the accurate measurement of these functions is a difficult task^21^.

It is therefore advantageous to use a reference signal, whose time-domain shape is well known, to eliminate ${{\bf{H}}}_{{\rm{prop}}}$ and ${{\bf{H}}}_{{\rm{EOS}}}$. A well-suited reference signal is the so-called shift current, mainly due to two reasons. First, the shift current dynamics is well understood; it is an all-optically induced current and results from the spatial shift of the electron charge during optical excitation of non-centrosymmetric semiconductors^22, 23^. Very often shift currents are regarded as the above-bandgap equivalent of rectification currents, with the latter denoting a difference frequency mixing process for below-bandgap photon energies. The main difference is that shift currents require real carriers, while optical rectification relies on virtual carriers. For above-bandgap photon excitation energies being energetically separated from exciton resonances or the bandgap energy itself, the shift current dynamics ${{\bf{j}}}_{{\rm{shift}}}$ follows the temporal intensity profile of the optical excitation ${{\bf{i}}}_{{\rm{opt}}}$, i.e., ${{\bf{j}}}_{{\rm{shift}}}\propto {{\bf{i}}}_{{\rm{opt}}}$. For such excitation photon energies, ballistic contributions, which coexist with shift currents and result from carrier scattering processes^24^, seem to play no significant role as recently confirmed by a detailed comparison between experiments and simulations which ignored any ballistic contribution^25^. Second, the shift current can be generated in one sample together with the AH currents without the need of interchanging any samples. To observe both currents, we used (110)-oriented bulk GaAs with the [001] axis parallel to the magnetic field. In this geometry no other (magneto-) photocurrent, like the circular bulk magneto-photocurrent, is allowed^17^. In analogy to Eq. (1) we can express the THz emission from the shift current as ${{\bf{E}}}_{{\rm{s}}{\rm{h}}{\rm{i}}{\rm{f}}{\rm{t}}}\propto {{\bf{H}}}_{{\rm{p}}{\rm{r}}{\rm{o}}{\rm{p}}}{{\bf{H}}}_{{\rm{E}}{\rm{O}}{\rm{S}}}i\omega {{\bf{J}}}_{{\rm{s}}{\rm{h}}{\rm{i}}{\rm{f}}{\rm{t}}}$. Comparing this equation with Eq. (1), we obtain for the AH current:

2 $${{\bf{j}}}_{{\rm{AH}}}={F}^{-1}\{{{\bf{I}}}_{{\rm{opt}}}\frac{{{\bf{E}}}_{{\rm{AH}}}}{{{\bf{E}}}_{{\rm{shift}}}}{\bf{R}}\},$$

with ${F}^{-1}$ denoting the inverse Fourier transformation. Here, we have considered the above mentioned relation ${{\bf{J}}}_{{\rm{shift}}}\propto {{\bf{I}}}_{{\rm{opt}}}$ (${{\bf{I}}}_{{\rm{opt}}}$ is obtained from an autocorrelation measurement) and ignored the scaling factor between the two quantities, as we are only interested in the time-domain shape of ${{\bf{j}}}_{{\rm{AH}}}$ but not in its amplitude. Morover, we have also introduced a regularization filter **R** (see methods), as it is necessary to suppress noise resulting from the deconvolution $(\frac{{{\bf{E}}}_{{\rm{AH}}}}{{{\bf{E}}}_{{\rm{shift}}}})$ described by Eq. (2).

The measurements of ${{\bf{e}}}_{{\rm{AH}}}$ and ${{\bf{e}}}_{{\rm{shift}}}$ have been carried out as follows. The samples have been excited at normal incidence with circularly polarized femtosecond pulses and a magnetic field along the $[001]$ or $[00\bar{1}]$ direction. With the dependence of ${{\bf{j}}}_{{\rm{AH}}}$ and ${{\bf{j}}}_{{\rm{shift}}}$ on the helicity of the excitation and the direction of the magnetic field, see Table 1, ${{\bf{e}}}_{{\rm{AH}}}$ and ${{\bf{e}}}_{{\rm{shift}}}$ have been extracted from the following measurements:

3 $${{\bf{e}}}_{{\rm{AH}}}=({{\bf{e}}}_{1}-{{\bf{e}}}_{2}-{{\bf{e}}}_{4}+{{\bf{e}}}_{5})/4,$$

4 $${{\bf{e}}}_{{\rm{shift}}}=({{\bf{e}}}_{3}+{{\bf{e}}}_{6})/2.$$

**Table 1:** Dependence of the shift current and AH current being directed along the [001] axis in (110)-oriented GaAs on the helicity of the optical polarization (**E**_opt_) and magnetic field (**B**) direction along the [001] axis.

Measurement	**E**_opt_	**B**	**j**_shift_	**j**_AH_
$${{\bf{e}}}_{1}$$				
$${{\bf{e}}}_{2}$$				
$${{\bf{e}}}_{3}$$				
$${{\bf{e}}}_{4}$$				
$${{\bf{e}}}_{5}$$				
$${{\bf{e}}}_{6}$$				

It has to be emphasized that during these measurements only the helicity of the optical excitation and the direction of the magnetic field have been changed according to Table 1. All other experimental conditions were absolutely identical.

## Results

**Figure 2:**
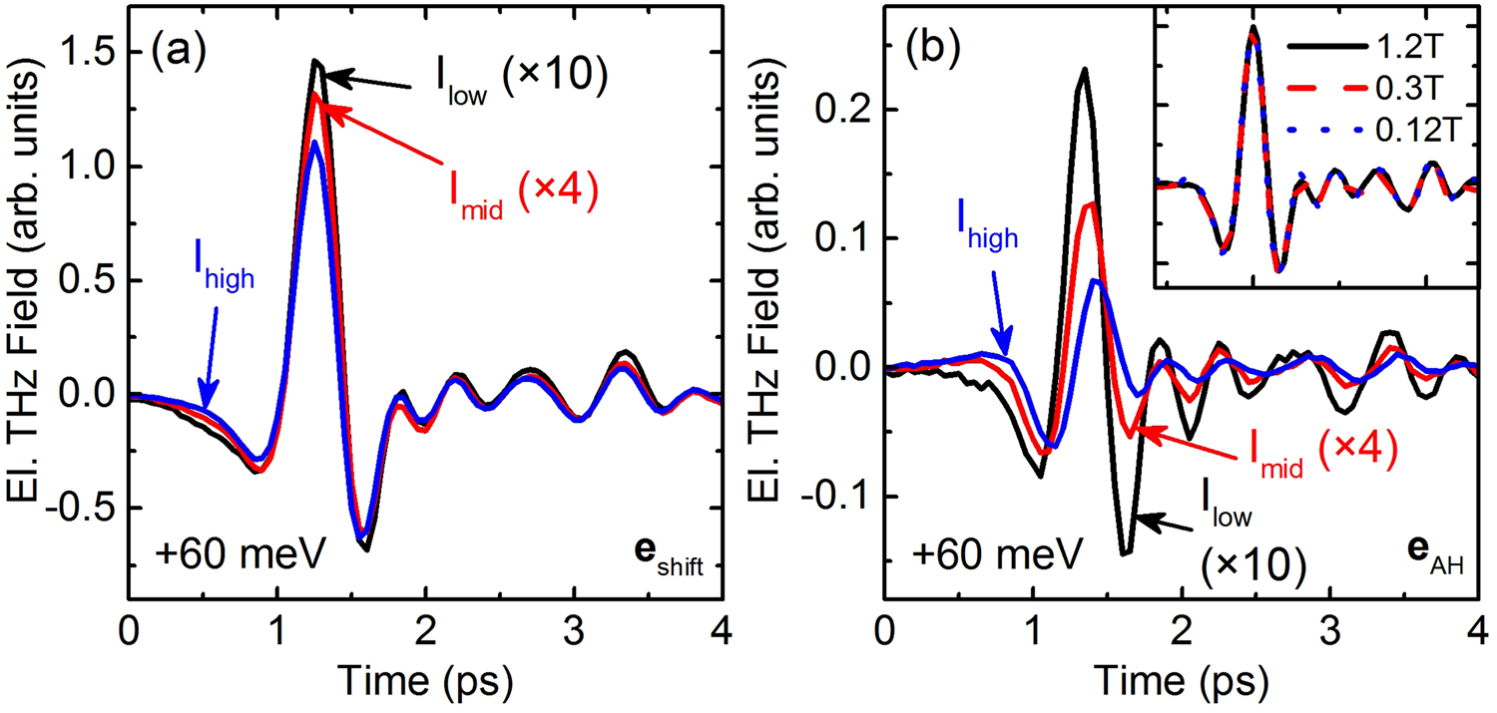
Measured THz traces emitted from shift currents (**a**) and AH currents (**b**) for three different optical peak intensities: *I*_low_ = 13 MW/cm^2^ (~4 × 10^16^ cm^−3^), *I*_mid_ = 32.5 MW/cm^2^ (~10^17^ cm^−3^), and *I*_high_ = 130 MW/cm^2^ (~4 × 10^17^ cm^−3^) with the number in the brackets denoting the optically induced carrier density. The THz traces have been scaled according to the optical intensity, such that an amplitude decrease denotes a sublinear intensity dependence. Inset: Measured THz traces emitted from AH currents for different magnetic fields but fixed optical intensity. The THz traces have been scaled according to the magnetic field. The excitation photon energy was 1.476 eV, i.e., 60 meV above the bandgap of GaAs.

Using Eqs (3) and (4), ${{\bf{e}}}_{{\rm{shift}}}$ and ${{\bf{e}}}_{{\rm{AH}}}$ have been extracted for three different pump intensities, see Fig. 2(a) and (b), respectively. The THz traces have been scaled to account for the different optical intensities. Therefore, the amplitude of the THz traces will be identical if they linearly depend on the optical intensity. This linearity is almost satisfied by ${{\bf{e}}}_{{\rm{shift}}}$, as expected from a perturbative second-order approach^22, 25^. Yet, more important than nonlinearities, which have been known for a long time^26^, is that the measurements confirm the intensity-independent shape of ${{\bf{e}}}_{{\rm{shift}}}$. In contrast, ${{\bf{e}}}_{{\rm{AH}}}$ shows a significantly larger nonlinear dependence of its amplitude on the optical intensity and the shape of the THz traces depends on the intensity, too. All global maxima of ${{\bf{e}}}_{{\rm{AH}}}$ occurring at approximately 2.2 ps appear slightly later in time as compared to the maxima of ${{\bf{e}}}_{{\rm{shift}}}$ and the absolute position of the maxima of ${{\bf{e}}}_{{\rm{AH}}}$ shifts to earlier times for a decrease of the optical intensity. The most prominent change of the shape results from a decrease of the second minimum of ${{\bf{e}}}_{{\rm{AH}}}$ for an increase of the optical intensity. The inset of Fig. 2(b) shows THz traces of the AH currents for three different magnetic fields and the same optical excitation intensity. The THz traces have been normalized by the magnetic field and perfectly overlap, proving a linear scaling of not only the AH currents’ amplitude but also the dynamics on the applied magnetic field. This observation additionally underlines the importance of time-resolved measurement techniques revealing for the first time the independence of AH currents’ dynamics on the magnetic field.

**Figure 3:**
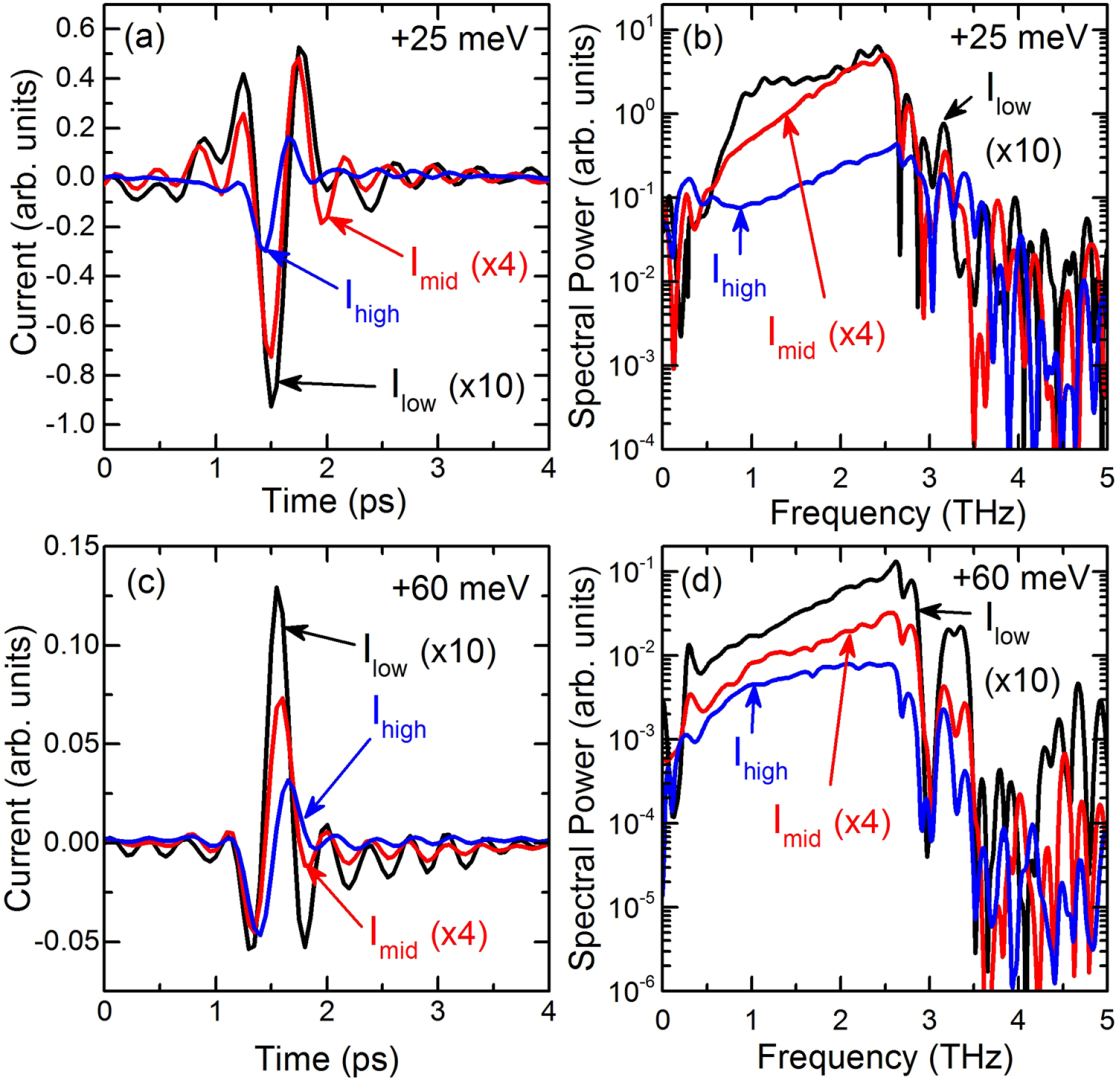
Extracted shapes of AH currents for three different optical intensities and 2 different photon energies. (**a**) 1.442 eV, i.e., 25 meV above the bandgap of GaAs, (**c**) 1.476 eV, i.e., 60 meV above the bandgap of GaAs. (**b**,**d**) Spectral amplitudes of the time-domain traces of (**a**,**c**), respectively. As in Fig. 2 the time-domain waveforms have been scaled according to the optical excitation intensity and the frequency-domain spectra have been calculated from theses scaled time-domain waveforms.

The analysis of ${{\bf{e}}}_{{\rm{AH}}}$ already shows that the dynamics of ${{\bf{j}}}_{{\rm{AH}}}$ depend on the optical excitation intensity and, thus differ from the dynamics of ${{\bf{j}}}_{{\rm{shift}}}$. We now employ Eq. (2) to obtain an estimation of the temporal shape of ${{\bf{j}}}_{{\rm{AH}}}$. The resulting time traces are shown in Fig. 3(a) and (c) for two different excitation photon energies: 1.442 eV and 1.476 eV, corresponding to an energy of 25 meV and 60 meV, respectively, above the bandgap of GaAs at room temperature. At high excitation intensities we obtain a current pulse with a single-cycle oscillation for both photon energies under study. With decrease in the excitation intensity the shape of ${{\bf{j}}}_{{\rm{AH}}}$ changes significantly. A sinc-like waveform with inverted peaks for 1.442 eV and 1.476 eV occurs indicating a current reversal. Thus, in addition to the excitation intensity dependence we also obtain a strong dependence of ${{\bf{j}}}_{{\rm{AH}}}$ on excitation photon energy with a current reversal between the two photon energies for low excitation intensities. It should be noted that especially for ${I}_{{\rm{low}}}$ and ${I}_{{\rm{mid}}}$ the current transients show small oscillations at early and late times. This behavior corresponds to an abrupt frequency cutoff of the corresponding spectra, which is also supported by Fig. 3(b) and (d). The frequency cutoff arises due to filtering of the THz traces by the transfer functions ${{\bf{H}}}_{{\rm{prop}}}$ and ${{\bf{H}}}_{{\rm{EOS}}}$, see Eq. (1). This filtering leads to a loss of information and obviously even the regularization filter **R** in Eq. (2) cannot recover the lost information.

Before discussing possible reasons for the above-mentioned dependences, we would like to comment on the validity of the data. Both, ${{\bf{e}}}_{{\rm{AH}}}$ and ${{\bf{e}}}_{{\rm{shift}}}$ were measured under the same experimental conditions, with the amplitude of ${{\bf{e}}}_{{\rm{shift}}}$ being approximately ten times larger than the amplitude of ${{\bf{e}}}_{{\rm{AH}}}$. Having these different THz amplitudes in mind one has to be cautious to correctly separate the current contributions from each other. Yet, there is a simple argument showing that the extraction of ${{\bf{j}}}_{{\rm{AH}}}$ is not influenced by ${{\bf{j}}}_{{\rm{shift}}}$. We clearly see that ${{\bf{e}}}_{{\rm{AH}}}$ has a stronger sublinear power dependence than ${{\bf{e}}}_{{\rm{shift}}}$. Thus, any leakage of ${{\bf{e}}}_{{\rm{shift}}}$ into ${{\bf{e}}}_{{\rm{AH}}}$ would be more prominent at larger intensities than at smaller intensities. However, we experimentally observe that the shape of ${{\bf{e}}}_{{\rm{AH}}}$ and ${{\bf{e}}}_{{\rm{shift}}}\,$(as well as ${{\bf{j}}}_{{\rm{AH}}}$ and ${{\bf{j}}}_{{\rm{shift}}}$) are more similar at small intensities than at large intensities (compare Fig. 2), proving that the extraction of ${{\bf{j}}}_{{\rm{AH}}}$ is not influenced by an improperly suppressed contribution of ${{\bf{j}}}_{{\rm{shift}}}$.

## Discussion

The extraction of the dynamics of ${{\bf{j}}}_{{\rm{AH}}}$ has produced three new results: (i) a complex bipolar shape of the current, (ii) a strong dependence of the temporal shape on the optically excited carrier density, and (iii) a strong dependence of the temporal shape on the photon energy corresponding to a current reversal between two photon energies at small carrier densities. We strongly believe that the only explanation for these complex dynamics is the interaction between several competing microscopic contributions with different dependences on photon energy and carrier density. While previous reviews^2, 9^ provide extensive information about intrinsic and extrinsic contributions in the steady state case, ultrafast temporal dynamics and, in particular, the temporal differences between the intrinsic and extrinsic dynamic responses have only been addressed in a recent publication^16^. However, the separation between intrinsic and extrinsic effects was accomplished without a magnetic field and at low temperatures where energy and spin relaxation were considerably longer than the optical pulse width. In the present study, these conditions were not met and, consequently, the separation will not work. The main goal of the following discussion is to bring up possible contributions to ${{\bf{j}}}_{{\rm{AH}}}$ rather than to undoubtedly identify these contributions.

We start with the expression for the total current, which also includes ${{\bf{j}}}_{{\rm{AH}}}$, given by ${\bf{j}}(t)=-e\,\overline{Tr}\{{\bf{v}}\rho (t)\}$, with $\rho (t)$ denoting the density matrix, and $\overline{Tr}$ expressing the momentum-space average of the trace. The time evolution of $\rho$ is obtained from the semiconductor Bloch equations^27^ and the microscopic velocity matrix **v** is defined as:

5 $${\bf{v}}={{\bf{v}}}_{{\rm{n}}}+{{\bf{v}}}_{{\rm{sj}}}+\frac{e}{\hslash }({{\bf{E}}}_{{\rm{sf}}}+{\bf{v}}\times {\bf{B}})\times {\boldsymbol{\Omega }}.$$

Here, ${{\bf{v}}}_{{\rm{n}}}$ is the normal velocity given by the energy dispersion. The side jump velocity is defined by ${{\bf{v}}}_{{\rm{sj}}}$. It is one of the origins of extrinsic effects and occurs due to carrier-impurity or carrier-phonon scattering^10, 28^. The other extrinsic effects result from ${{\bf{v}}}_{{\rm{n}}}$ in combination with electric and magnetic field influences on $\rho (t)$ ^10^. Intraband motions are manipulated by the built-in surface field $({{\bf{E}}}_{{\rm{sf}}})$ and the applied magnetic field $({\bf{B}})$ both of which in combination with the Berry curvature $({\boldsymbol{\Omega }})$ result in the intrinsic contribution.

As mentioned above, one of the important observations of our experiments is the complex time-domain shape of ${{\bf{j}}}_{{\rm{AH}}}$. It is very likely that this finding is linked to current contributions with different onset and decay times. A current onset time exists since after carrier excitation the spin-Hall angle needs to be formed. This is done by rotation of the carrier distribution and the carrier spin in the external magnetic field with a cyclotron frequency and Lamor frequency of ${{\rm{\omega }}}_{c}/2\pi \approx 0.4\,{\rm{THz}}$ and ${{\rm{\omega }}}_{l}/2\pi \approx 4\,{\rm{GHz}}$ ^29^, respectively, for electrons in GaAs and a magnetic field of 1 T. Since ${{\rm{\omega }}}_{c}\gg {{\rm{\omega }}}_{l}$ the Lamor precession can be neglected as compared to the Hall rotation, but the cyclotron frequencies for heavy and light holes differ from the electron cyclotron frequency. In addition to these different current onset times, the current decay for both, intrinsic and extrinsic effects is typically influenced by a combination of spin and momentum/energy relaxation^16^. The different time scales of these processes involving electrons as well as heavy- and light holes, might explain the complex time-domain shape of ${{\bf{j}}}_{{\rm{AH}}}$ with positive and negative contributions.

**Figure 4:**
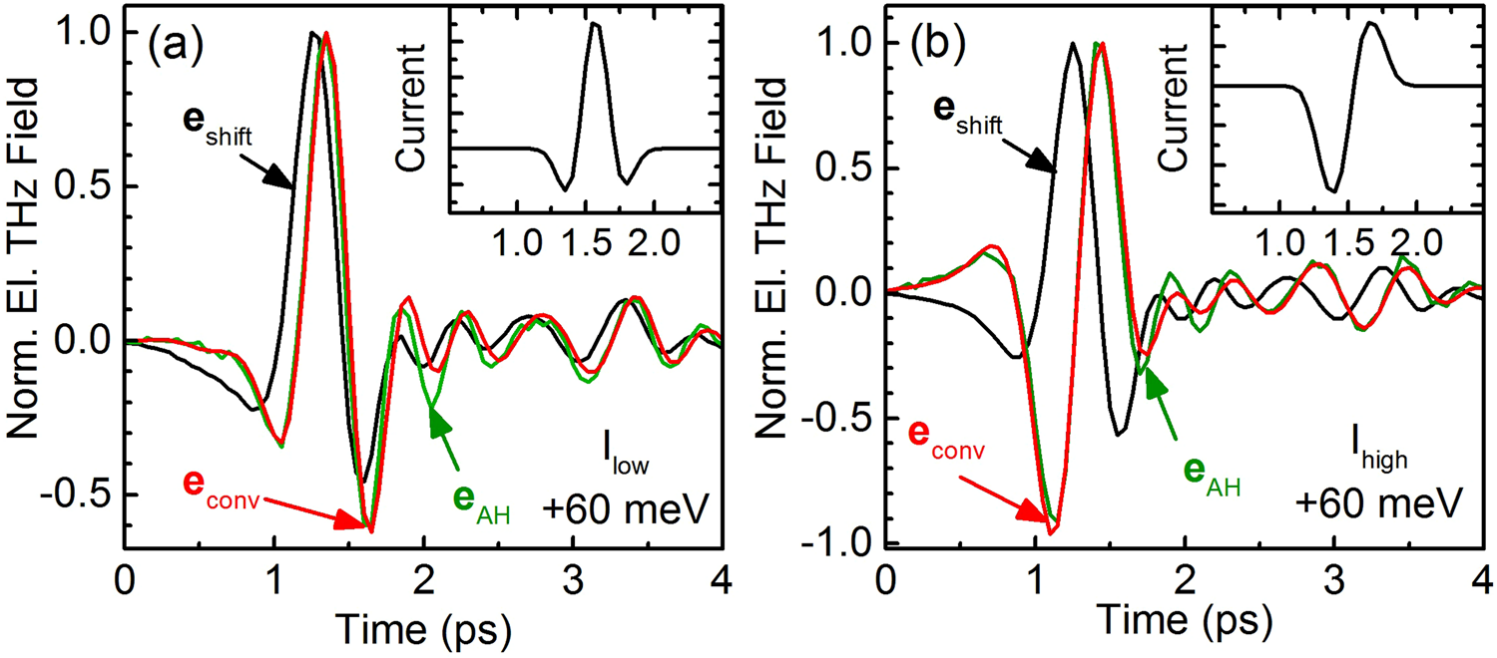
Measured and normalized THz traces emitted from shift currents (${{\bf{e}}}_{{\rm{shift}}}$, black lines) and AH currents (${{\bf{e}}}_{{\rm{AH}}}$, green lines) for two different optical peak intensities: *I*_low_ = 13 MW/cm^2^ (**a**) and *I*_high_ = 130 MW/cm^2^ (**b**) and for an excitation photon energy of 1.476 eV, i.e., 60 meV above the bandgap of GaAs. The red lines (${{\bf{e}}}_{{\rm{conv}}}$) denote the THz traces resulting from a convolution of the current pulses shown in the insets with an inverse Fourier transformation of ${{\bf{E}}}_{{\rm{shift}}}/{{\bf{I}}}_{{\rm{opt}}}$.

It should be emphasized that the bipolar shape of ${{\bf{j}}}_{{\rm{AH}}}$ does not result from the regularization process. This can easily be demonstrated by constructing bipolar current shapes, which resemble the extracted shapes of ${{\bf{j}}}_{{\rm{AH}}}$ but have no additional oscillation-like features, see insets of Fig. 4(a) and (b). Multiplying the frequency-domain representation of these constructed current pulses with ${{\bf{E}}}_{{\rm{shift}}}/{{\bf{I}}}_{{\rm{opt}}}$ and performing an inverse Fourier transformation we obtain THz traces ${{\bf{e}}}_{{\rm{conv}}}$, which resemble ${{\bf{e}}}_{{\rm{AH}}}$ very well, see Fig. 4(a) and (b). Since the resulting THz traces ${{\bf{e}}}_{{\rm{conv}}}$ were obtained without regularization, it becomes evident that the bipolar shapes of ${{\bf{j}}}_{{\rm{AH}}}$ in Fig. 3(a) and (c) are real and not an artifact appearing because of the regularization.

To explain photon energy dependent features of ${{\bf{j}}}_{{\rm{AH}}}$ we examine various microscopic origins. First, it is well known that ${\boldsymbol{\Omega }}$ and/or ${\bf{v}}$ are momentum dependent^30^, such that the expression $({{\bf{E}}}_{{\rm{sf}}}+{\bf{v}}\times {\bf{B}})\times {\boldsymbol{\Omega }}$ will depend on the excitation photon energy. Moreover, the two individual contributions ${{\bf{E}}}_{{\rm{sf}}}\times {\boldsymbol{\Omega }}$ and $({\bf{v}}\times {\bf{B}})\times {\boldsymbol{\Omega }}$ will have a different dependence on the excitation photon energy. In addition to these intrinsic effects we cannot fully exclude that carrier-phonon scattering^28^ also influences the excitation photon energy dependence of ${{\bf{j}}}_{{\rm{AH}}}$. While carriers with a small excess energy above the bandgap cannot emit phonons, this bottleneck is lifted for larger excess energies. Another indication that extrinsic effects influence ${{\bf{j}}}_{{\rm{AH}}}$ is obtained from the density dependence. While the carrier density is supposed to have small effects on intrinsic contributions, it is well known that carrier scattering effects are largely affected by the carrier density^31, 32^. Therefore, it is very likely that various microscopic mechanisms, which compete against each other, are at the origin of the complex current shapes and the photon energy dependence of the current shape.

In conclusion, we presented a concept to non-invasively investigate the dynamics of the AH effect in a simple THz time-domain spectroscopy experiment. While our results show that the AH effect consists of different mechanisms occurring in the femtosecond time regime at room temperature, we could not identify certain microscopic contributions to ${{\bf{j}}}_{{\rm{AH}}}$. This requires a more elaborate theoretical treatment involving influences of phonons, impurities, carriers, and the photon energy dependence of the Berry curvature and additional measurements at low temperatures, both of which is beyond the scope of this work. Despite these limitations we believe that our time-resolved measurements of ${{\bf{j}}}_{{\rm{AH}}}$ are an essential step towards full time resolution of spin Hall and AH effects being a prerequisite for a better understanding of the involved microscopic phenomena and for using these effects in ultrafast spintronic applications.

## Methods

### Experimental details

As light source a 76 MHz Ti:Sa laser with a temporal and spectral pulse width of 180 fs and 10 meV, respectively, was used. The pump beam was focused on a 450 µm thick (110)-orientated GaAs wafer with a spot diameter of 220 µm and a peak intensity of up to 130 MW cm^−2^. The intensity of the excitation was controlled by a half-wave plate and a linear polarizer, while a quarter-wave plate allowed us to control the optical polarization state. Two off-axis parabolic mirrors were employed to collect the THz radiation emitted from the backside of the sample and to focus it onto a 1 mm thick ZnTe crystal. Here the THz radiation is collinearly overlapped with a femtosecond probe beam obtained from the same laser system. The electric-field induced change of the probe-beam polarization is analyzed and taken as a measure of the electric field strength. For this purpose, a typical electro-optic sampling configuration is employed, in which the time-delay between the pump and probe pulses is changed in order to sample the shape of the THz traces. With a THz polarizer and proper orientation of the ZnTe crystal in the setup we ensured exclusive detection of THz radiation from currents flowing (anti-) parallel to the magnetic field. Currents resulting from the surface electric field and Hall rotation were suppressed. The magnetic field was obtained by an electro-magnet with field strengths ranging between ±1.2 T at the sample position. All experiments were done at room temperature.

### Transfer functions of THz propagation and detection

The measured THz traces differ from the time-derivation of the current transients mainly due to two reasons^19, 20^. First, the propagation from the sample towards the ZnTe detection crystal leads to changes of the time-domain shape of the THz pulses. This occurs because of (i) Fabry-Perot effects in the sample, (ii) atmospheric effects, and (iii) effects due to collecting and focusing of the THz radiation. The corresponding transfer function is denoted by ${{\bf{H}}}_{{\rm{prop}}}$. Second, the EOS process leads to an additional change of the THz pulse shape. This is due to (i) the mismatch between the optical group velocity and the THz phase velocity in the EOS crystal, (ii) phonon resonances in the crystal, and (iii) spatial overlap between optical and THz pulses in the crystal. The corresponding transfer function is denoted by ${{\bf{H}}}_{{\rm{EOS}}}$.

### Regularization

The division of complex spectra given by Eq. (2) corresponds to a time-domain deconvolution. A deconvolution of signals containing noise is an ill-posed problem and, thus, requires regularization^33^. Expressing the deconvolution by ${\bf{H}}={{\bf{X}}}_{2}/{{\bf{X}}}_{1}$, one typically applies a regularization filter **R** such that ${\bf{H}}\to {\bf{H}}{\bf{R}}$. In this work a Tikhonov regularization filter has been used given by ${\bf{R}}={|{{\bf{X}}}_{1}|}^{2}/({|{{\bf{X}}}_{1}|}^{2}+{\rm{\gamma }}{|{\bf{M}}|}^{2})$, where γ and **M** are appropriate regularization parameter and operator, respectively. As regularization operator the identity function has been employed and the regularization parameter has been determined using the L-curve method^34^.

### Data Availability

The datasets generated during and/or analysed during the current study are available from the corresponding author on reasonable request.
